# Role of Nicotine Dependence on the Relationship between Variants in the Nicotinic Receptor Genes and Risk of Lung Adenocarcinoma

**DOI:** 10.1371/journal.pone.0107268

**Published:** 2014-09-18

**Authors:** Tung-Sung Tseng, Jong Y. Park, Jovanny Zabaleta, Sarah Moody-Thomas, Melinda S. Sothern, Ted Chen, David E. Evans, Hui-Yi Lin

**Affiliations:** 1 Behavioral and Community Health Sciences, School of Public Health and Stanley S. Scott Cancer Center, Louisiana State University Health Sciences Center, New Orleans, LA, United States of America; 2 Department of Cancer Epidemiology, H. Lee Moffitt Cancer Center and Research Institute, Tampa, FL, United States of America; 3 Department of Pediatrics and Stanley S. Scott Cancer Center, Louisiana State University Health Sciences Center, New Orleans, LA, United States of America; 4 Department of Global Community Health and Behavioral Sciences, Tulane University, New Orleans, LA, United States of America; 5 Department of Health Outcomes and Behavior, H. Lee Moffitt Cancer Center and Research Institute, Tampa, FL, United States of America; 6 Department of Biostatistics and Bioinformatics, H. Lee Moffitt Cancer Center and Research Institute, Tampa, FL, United States of America; Yale University, United States of America

## Abstract

Several variations in the nicotinic receptor genes have been identified to be associated with both lung cancer risk and smoking in the genome-wide association (GWA) studies. However, the relationships among these three factors (genetic variants, nicotine dependence, and lung cancer) remain unclear. In an attempt to elucidate these relationships, we applied mediation analysis to quantify the impact of nicotine dependence on the association between the nicotinic receptor genetic variants and lung adenocarcinoma risk. We evaluated 23 single nucleotide polymorphisms (SNPs) in the five nicotinic receptor related genes (*CHRNB3, CHRNA6, and CHRNA5/A3/B4*) previously reported to be associated with lung cancer risk and smoking behavior and 14 SNPs in the four ‘control’ genes (*TERT*, *CLPTM1L, CYP1A1*, and *TP53*), which were not reported in the smoking GWA studies. A total of 661 lung adenocarcinoma cases and 1,347 controls with a smoking history, obtained from the Environment and Genetics in Lung Cancer Etiology case-control study, were included in the study. Results show that nicotine dependence is a mediator of the association between lung adenocarcinoma and gene variations in the regions of *CHRNA5/A3/B4* and accounts for approximately 15% of this relationship. The top two *CHRNA3* SNPs associated with the risk for lung adenocarcinoma were rs1051730 and rs12914385 (p-value = 1.9×10^−10^ and 1.1×10^−10^, respectively). Also, these two SNPs had significant indirect effects on lung adenocarcinoma risk through nicotine dependence (p = 0.003 and 0.007). Gene variations rs2736100 and rs2853676 in *TERT* and rs401681 and rs31489 in *CLPTM1L* had significant direct associations on lung adenocarcinoma without indirect effects through nicotine dependence. Our findings suggest that nicotine dependence plays an important role between genetic variants in the *CHRNA5/A3/B4* region, especially *CHRNA3,* and lung adenocarcinoma. This may provide valuable information for understanding the pathogenesis of lung adenocarcinoma and for conducting personalized smoking cessation interventions.

## Introduction

Lung cancer was the second leading cause of cancer incidence (14%) and the first leading cause of cancer deaths (27%) for Americans in 2014 [1]. There are two major histological categories for lung cancer: small cell lung cancer (SCLC) and non-small cell lung cancer (NSCLC). NSCLC is classified into three subtypes: adenocarcinoma (ADC), squamous cell carcinoma (SQC), and large cell carcinoma (LC). Worldwide, ADC is the most common type of lung cancer, with an increasing trend of incidence over time for both males and females [2]. Most lung cancer patients are diagnosed at advanced stages, and the 5-year survival rate for these patients is less than 10% [3], [4]. Despite its negative impact on public health, effective early detection tools for lung cancer are still under development. Chest X-ray screening with or without sputum cytologic analysis have shown no reduction in lung-cancer mortality based on several randomized trials [5]. In another large scale clinical trial with more than 53,000 participants, low-dose computed tomography (CT) scans can reduce mortality from lung cancer by 20% relative to chest X-ray screening among the high-risk group with a history of heavy smoking. However, CT scan screening showed high false-positive results (96%) [6]. Thus, there is an urgent need for identifying additional biomarkers in order to improve prediction accuracy of lung cancer. While exploring biomarkers of lung cancer, smoking needs to be taken into consideration.

Smoking, which is a well-known modifiable behavior, is the leading risk factor of lung cancer. The impact of smoking on lung cancer is enormous. Both quantity and duration of smoking increase lung cancer risk [7]. The historical cigarette smoking prevalence can be used to predict the trend of lung cancer incidence in both men and women based on a study using 1920–1990 data [8]. A similar trend impact of smoking is also shown in lung ADC [9]. Smoking is generally involved in the development of ADC [10], although the risk is less for lung ADC than for SQC and SCLC [11].

Although smoking is the most important behavioral factor of lung cancer, the impact of smoking on the associations between variants in nicotine related genes and lung cancer is still understudied. In attempts to elucidate these relationships, we have used mediation analysis in this study to quantify the mediation effect of nicotine dependence. *CHRNA5/A3/B4, CHRNB3,* and *CHRNA6* genes are nicotinic receptor genes, encoding nicotine acetylcholine receptor subunits. The nicotinic cholinergic receptor subunits expressed in the human brain form various types of functional receptors by different subunit composition and play a vital role in modulation of dopaminergic function and sensitivity to nicotine [12]. These five genes have been reported to be associated with both smoking behavior and lung cancer risk [13], [14]. *CHRNB3* and *CHRNA6* are in the chromosome 8p11 region. Single nucleotide polymorphisms (SNPs) in *CHRNB3* were associated with nicotine dependence [15] and cigarettes smoked per day [16]. Variations in *CHRNA6* are associated with nicotine dependence [15] and tobacco phenotypes [17]. Based on the National Human Genome Research Institute (NHGRI) GWAS Catalog [18], SNPs in *CHRNA5/A3/B4* are associated with overall lung cancer, lung ADC, and smoking; SNPs in *CHRNB3* are associated with cigarettes smoked per day.

Another four genes (*TERT, CLPTM1L, CYP1A1*, and *TP53*) are associated with lung cancer risk. In several GWA studies, SNPs in *TERT* and *CLPTM1L* are associated with overall lung cancer and lung ADC [18]. Genetic variants of the *CYP1A1* exon7 are reported to be associated with lung cancer in the overall population, especially in Asians, Caucasians, females, and smokers [19]. The *CYP1A1* polymorphisms [Ile462Val (rs1048943) and T6235C (rs4646903)] are associated with lung cancer risk, especially for lung SQC in Asian populations [20]. Another meta-analysis indicates that TP53 codon 72 and intron 6 (rs1625895) polymorphisms are associated with lung cancer risk [21].

There was a limited number of related mediation studies to evaluate the impact of smoking (number of cigarettes smoked per day or smoking pack-year) on the relationship between variants (especially rs1051730) in CHRNA5/A3/B4 and overall lung cancer or chronic obstructive pulmonary disease (COPD) [22]–[24]. However, nicotine dependence and its mediation impact on lung ADC remain unexplored. Lung cancer is a heterogeneous disease so it is important to evaluate specific histological categories of lung cancer separately in genetic studies. In addition, nicotine dependence, which largely contributes to persistent smoking, has a large impact on lung cancer. Previous studies show that 60–70% of the variance in smoking persistence and nicotine dependence result from genetic impact [25]–[27]. In our study, nicotine dependence was measured using the Fagerstrom Test [28], which correlates well with various nicotine withdrawal symptoms, level of smoking urges, and self-rated addition [29]–[31]. Therefore, the **objective** was to characterize the mediation effects of nicotine dependence on the relationship between genetic variants in the five nicotinic receptor genes (*CHRNA5/A3/B4, CHRNB3,* and *CHRNA6*) and lung ADC risk among ever smokers. In order to evaluate robustness of the mediation analysis, we also included four ‘control’ genes (*TERT*, *CLPTM1L, CYP1A1*, and *TP53*), which are not the nicotinic receptor genes and were not reported in the smoking GWA studies.

## Methods

### Study population and measurements

A total of 661 lung adenocarcinoma (ADC) cases and 1,347 controls with a smoking history obtained from the Environment and Genetics in Lung Cancer Etiology (EAGLE) case-control study were included in this study. Due to the heterogeneity of lung cancer, we focused only on the most common histology type: Lung ADC. This dataset is a part of the GENEVA/GEI lung cancer and smoking GWS dataset (dbGaP accession number: phs000093.v2.p2). DNA samples were genotyped on the Illumina HumanHap550v3_B BeadChips (Illumina, San Diego, CA, USA). SNPs with minor allele frequencies (≤5%), completion rates (≤95%), or a p-value of Hardy-Weinberg equilibrium in controls<10^−7^ were excluded [32]. Included were 37 SNPs in nine candidate genes (*CHRNB3, CHRNA6, CHRNA5/A3/B4, TERT*, *CLPTM1L, CYP1A1*, and *TP53*).

All participants, enrolled in Italy between 2002 and 2005, were self-identified as White. We included only ever smokers, which was defined as those who smoked at least 100 cigarettes in their entire lifetime. Ever smokers were chosen because (1) the mechanism of lung cancer development may be different for ever smokers and never smokers [13], and (2) ever smokers are commonly used as the targeted population for early detection of lung cancer. Nicotine dependence was measured using the 6-item Fagerstrom Test [28], with a score range of 0 to 10. Higher scores indicated greater nicotine dependence. For former smokers who quit smoking ≥6 months previously, nicotine dependence at the time in which they smoked the most was reported. Pack-year, estimated by number of cigarette packs (20 cigarettes = 1 pack) times years of smoking, was also included. The details of this study are presented at http://www.ncbi.nlm.nih.gov/projects/gap/cgi-bin/study.cgi?study_id=phs000093.v2.p2.

### Statistical analysis

Participants’ demographic and smoking characteristics by disease status were compared using t-tests for continuous variables and chi-square tests for categorical variables. SNPs were treated as an additive model with a minor allele count. The binary nicotine dependence status (high/low) was used in the analyses. High nicotine dependence was defined as a Fagerstrom score ≥6, which is commonly used in other studies [33]–[36]. Mediation analyses based on the Baron and Kenny method [37] were performed. In these analyses, the SNP was the independent variable; lung cancer was the outcome variable; and nicotine dependence was the proposed mediator. In our mediation analysis, logistic regressions were applied for modeling the nicotine dependence and lung cancer risk. The mediated effect is robust in terms of the logistic assumption [38]. All models were adjusted for age and gender. The odds ratios (ORs) per minor allele were calculated.

**Figure 1:**
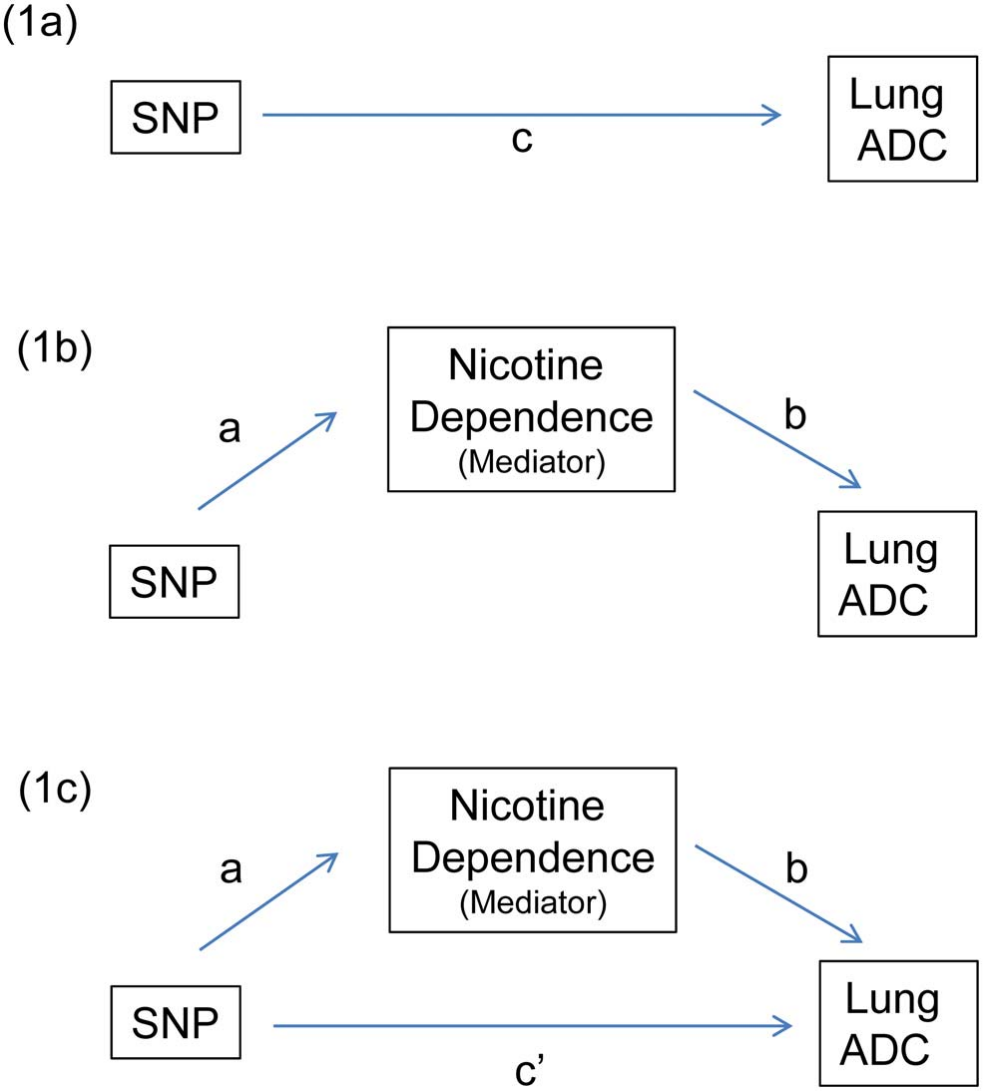
Role of nicotine dependence on the association between SNPs and lung adenocarcinoma (ADC) risk. The SNP associated with lung ADC risk are (1a) total effect without mediator, (1b) indirect-only effect, and (1c) with mediator. ***a*** is the logistic regression coefficient of nicotine dependence on an SNP. ***b*** is the coefficient on nicotine dependence, and ***c*** **′** denotes a direct effect, which is the coefficient on the SNP in a logistic regression of lung ADC with nicotine dependence and a given SNP. **c**, is the total effect, which is the logistic regression coefficient of lung ADC on a given SNP without controlling nicotine dependence. **a**×**b** denotes an indirect effect.

For each SNP, the mediation analyses were based on the following three logistic models (Equations 1–3). For simplicity, the covariates of age and gender are not shown in the equations.
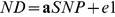
(1)

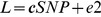
(2)

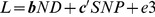
(3)Here, ND represents nicotine dependence, L represents lung cancer, and e1–e3 represent the error terms for each equation. In these equations and Figure 1, ***a*** was the coefficient of nicotine dependence on an SNP (Equation 1). The total effect, denoted as **c**, was the coefficient of lung cancer on an SNP without considering nicotine dependence (Equation 2). Using a logistic model of lung cancer with nicotine dependence and a given SNP (Equation 3), ***b*** was the coefficient on nicotine dependence, and ***c*** **′** was the coefficient on the SNP. The indirect effect of an SNP on lung cancer risk is defined as **a**×**b**, the direct effect as **c′**, and the total effect as ***c***. When the logistic regressions were applied for the binary mediator and/or binary outcome, the coefficients in the mediation analysis have different scales. Thus, standardized coefficients (**a_s_**, **b_s_**, **c_s_′**) are needed in order to make these coefficients comparable across models. The standardized coefficient was calculated by multiplying the original coefficient by the standard deviation of the same predictor variable in the model and then dividing by the standard deviation of the outcome variable [39]. The mediation proportion was calculated by **a_s_**×**b_s_**/(**a_s_**×**b_s_**+**c_s_′**). When **a_s_**×**b_s_** and **c_s_′** had the same direction (both negative or both positive), the relative indirect effect can be interpreted as the mediation proportion [40]. The indirect effect was evaluated using the Sobel test [41], [42]. The 95% bootstrap confidence intervals of the indirect effect (**a_s_**×**b_s_**) based on 2000 bootstrap samples are also presented. Linkage disequilibrium (LD) was measured using r^2^. The false discovery rate (FDR) q-values [43] were calculated for taking multiple comparisons into consideration. Associations with an FDR q-value <0.05 were considered statistically significant. Analyses were performed using SAS (SAS Institute, Cary, NC).

## Results

Samples from the 661 lung ADC patients and 1,347 controls in the GENEVA/GEI lung cancer and smoking study were analyzed. The distributions of age at diagnosis for cases or at review for controls were similar (Table 1). There were more female (20.3% vs. 15.1%, p = 0.004) and more current smokers (52.8% vs. 36.3%, p = <0.001) for the ADC patients than the controls. As expected, the ADC patients had significantly higher pack-year and higher nicotine dependence (mean score 4.5 vs. 2.8) than the controls. Over 30% of the ADC patients (vs. 13% controls) had more than 50 pack-years. Cigarette pack-year and nicotine dependence were highly correlated (Spearman correlation = 0.70).

**Table 1:** Participants’ characteristics by disease status.

Characteristics	Control (n = 1,347)	Adenocarcinoma(n = 661)	p-value^1^
	N (%)	N (%)	
Age			
< = 59	328 (24.4)	189 (28.6)	0.113
60–64	253 (18.8)	127 (19.2)	
65–69	307 (22.8)	144 (21.8)	
70–74	283 (21.0)	111 (16.8)	
Gender			
Male	1143 (84.9)	527 (79.7)	0.004
Female	204 (15.1)	134 (20.3)	
Smoking status			
Former	858 (63.7)	312 (47.2)	<.0001
Current	489 (36.3)	349 (52.8)	
Cigarette pack-year			
0.1–30	832 (61.8)	201 (30.4)	<.0001
31–50	343 (25.5)	253 (38.3)	
50+	172 (12.8)	207 (31.3)	
Nicotine dependence^2^			
Mean ± SD	2.8±2.5	4.5±2.5	
Low (<6)	1118 (83.0)	414 (62.6)	<.0001
High (≥6)	229 (17.0)	247 (37.4)	<.0001

1 t-test for continuous variables and chi-square test for categorical variables.

2 measured using the Fagerstrom Test, with a score range of 0 to 10. Higher scores indicated greater nicotine dependence. SD: standard deviation.

**Figure 2:**
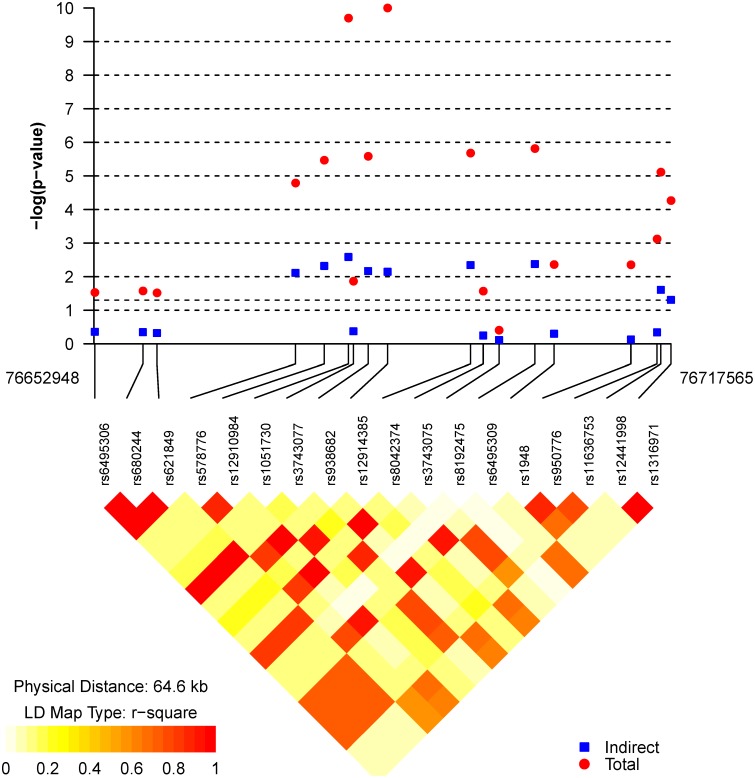
Genetic variants in *CHRNA5/A3/B4* associated with lung adenocarcinoma. Indirect effect: SNPs impact on lung adenocarcinoma through nicotine dependence Total effect: SNPs impact on lung adenocarcinoma without considering nicotine dependence.

Most of the variations in *CHRNA5/A3/B4* had significant total effects on lung ADC (Table 2 and Figure 2), and rs12441998 in *CHRNB4* was significantly associated with nicotine dependence (***a*** coefficient in Table 3). In this region, most of the SNPs in *CHRNA3* had significant indirect effects on lung ADC through nicotine dependence, and their relationship is shown in Figure 1c. Three SNPs in *CHRNA5* (rs6495306, rs680244 and rs621849), in complete LD (r^2^ = 0.99), had a significant total effect (p = 0.03, FDR q = 0.023) and direct effects (p = 0.04, FDR q = 0.034), but the indirect effect through nicotine dependence was not significant. Nine SNPs in *CHRNA3* had significant total and direct effects; seven of these had significant indirect effects on lung ADC through nicotine dependence. The top two SNPs associated with lung ADC (total effect) were with a strong LD (r^2^ = 0.93); these were rs1051730 (OR = 1.56 per T-allele, p = 1.9×10^−10^, FDR q = 1.5×10^−9^, and rs12914385 (OR = 1.56 per T-allele, p = 1.1×10^−10^, FDR q = 1.5×10^−9^). Their effects mediated through nicotine dependence were significant (p = 0.003 and 0.007, and FDR q-value = 0.023, and 0.023, respectively). The relative indirect effects for these top two SNPs were 13% and 11%, respectively. The mediation proportions of SNPs in *CHRNA3* were 11–17% (mean = 15.1%). The indirect effects and their 95% bootstrap confidence intervals were shown in Table S1. All five SNPs in *CHRNB4* had significant total and direct effects, and only two SNPs (rs12441998 and rs1316971) had a raw p-value <0.05 for the indirect effect. However, they became insignificant after considering multiple comparisons (FDR q-value = 0.056 and 0.072, respectively).

**Table 2:** Gene variations associated with lung adenocarcinoma in ever smokers.

					Total effect^1^		Direct effect^1^		IND	Med
Ch^1^	Gene	SNP^1^	A/a^2^	MAF^2^	OR (95% CI)^3^	p-value	OR (95% CI)^3^	p-value	p-value^4^	%^4^
5	TERT	rs2736122	C/T	0.22	1.02 (0.87–1.19)	0.849	1.01 (0.86–1.19)	0.868	0.889	-
		rs4975605	C/A	0.48	0.94 (0.83–1.07)	0.380	0.97 (0.85–1.11)	0.686	0.037	-
		rs2736100	G/T	0.44	0.76 (0.67–0.87)	**8.2×10^−5^** *	0.76 (0.66–0.88)	**1.2×10^−4^** *	0.405	-
		rs2853676	G/A	0.31	1.17 (1.02–1.34)	**0.029** *	1.17 (1.01–1.35)	**0.031** *	0.722	-
5	CLPTM1L	rs402710	C/T	0.31	0.99 (0.85–1.14)	0.851	0.98 (0.84–1.14)	0.774	0.690	-
		rs10073340	C/T	0.16	1 (0.83–1.2)	0.975	1 (0.83–1.2)	0.973	0.987	-
		rs401681	C/T	0.44	0.83 (0.72–0.95)	**0.007** *	0.84 (0.73–0.97)	**0.015** *	0.143	-
		rs31489	C/A	0.40	0.85 (0.74–0.97)	**0.018** *	0.85 (0.74–0.98)	**0.024** *	0.419	-
8	CHRNB3^#^	rs6474414	C/A	0.22	0.87 (0.74–1.02)	0.092	0.9 (0.76–1.06)	0.222	0.033	-
		rs7012713	C/T	0.03	0.84 (0.58–1.22)	0.351	0.85 (0.58–1.25)	0.416	0.512	-
8	CHRNA6^#^	rs892413	C/A	0.17	1 (0.84–1.19)	0.990	1 (0.84–1.2)	0.958	0.879	-
		rs16891604	C/A	0.08	1 (0.78–1.28)	0.990	1 (0.78–1.29)	0.986	0.908	-
		rs16891620	C/A	0.11	1.02 (0.83–1.26)	0.850	1.01 (0.81–1.25)	0.935	0.641	-
15	CYP1A1	rs4646421	C/T	0.11	1.22 (0.99–1.51)	0.056	1.16 (0.94–1.44)	0.161	0.020	-
		rs2470893	G/A	0.20	1.06 (0.9–1.25)	0.492	1.02 (0.86–1.21)	0.797	0.061	-
15	CHRNA5^#^	rs6495306	A/G	0.35	0.86 (0.75–0.98)	**0.030** *	0.86 (0.75–1)	**0.043** *	0.439	-
		rs680244	G/A	0.35	0.85 (0.74–0.98)	**0.027** *	0.86 (0.75–0.99)	**0.038** *	0.450	-
		rs621849	A/G	0.35	0.86 (0.75–0.99)	**0.030** *	0.86 (0.75–0.99)	**0.042** *	0.479	-
15	CHRNA3^#^	rs578776	C/T	0.27	0.71 (0.61–0.83)	**1.6×10^−5^** *	0.73 (0.63–0.86)	**1.6×10^−4^** *	**0.008** *	16.9
		rs12910984	A/G	0.24	0.68 (0.57–0.8)	**3.4×10^−6^** *	0.7 (0.59–0.83)	**4.1×10^−5^** *	**0.005** *	16.8
		rs1051730	C/T	0.41	1.56 (1.36–1.78)	**1.9×10^−10^** *	1.51 (1.31–1.74)	**6.9×10^−9^** *	**0.003** *	12.7
		rs3743077	G/A	0.35	0.84 (0.73–0.96)	**0.014** *	0.84 (0.73–0.97)	**2.0×10^−2^** *	0.422	-
		rs938682	T/C	0.23	0.67 (0.57–0.79)	**2.6×10^−6^** *	0.7 (0.59–0.83)	**2.8×10^−5^** *	**0.007** *	15.7
		rs12914385	C/T	0.44	1.56 (1.36–1.78)	**1.1×10^−10^** *	1.52 (1.33–1.75)	**2.4×10^−9^** *	**0.007** *	11.1
		rs8042374	A/G	0.24	0.67 (0.57–0.79)	**2.1×10^−6^** *	0.7 (0.59–0.82)	**2.6×10^−5^** *	**0.005** *	16.3
		rs3743075	G/A	0.31	0.85 (0.73–0.98)	**0.027** *	0.85 (0.73–0.99)	**0.034** *	0.565	-
		rs8192475	G/A	0.04	0.86 (0.61–1.22)	0.394	0.87 (0.61–1.24)	0.432	0.769	-
		rs6495309	C/T	0.23	0.66 (0.56–0.78)	**1.5×10^−6^** *	0.69 (0.58–0.82)	**2.0×10^−5^** *	**0.004** *	16.3
15	CHRNB4^#^	rs1948	C/T	0.30	0.81 (0.7–0.93)	**0.004** *	0.81 (0.69–0.94)	**0.006** *	0.502	-
		rs950776	T/C	0.30	0.81 (0.7–0.94)	**0.004** *	0.8 (0.69–0.93)	**0.005** *	0.745	-
		rs11636753	G/T	0.34	0.78 (0.68–0.9)	**0.001** *	0.78 (0.67–0.91)	**0.001** *	0.456	-
		rs12441998	A/G	0.22	0.68 (0.57–0.8)	**7.7×10^−6^** *	0.7 (0.59–0.83)	**4.7×10^−5^** *	0.025	-
		rs1316971	G/A	0.22	0.71 (0.6–0.84)	**5.4×10^−5^** *	0.72 (0.61–0.86)	**2.3×10^−4^** *	0.049	-
17	TP53	rs12951053	A/C	0.06	1.06 (0.82–1.38)	0.666	1.14 (0.87–1.48)	0.355	0.049	-
		rs2909430	A/G	0.17	1 (0.84–1.19)	0.975	1.01 (0.85–1.21)	0.909	0.529	-
		rs8079544	C/T	0.03	0.78 (0.52–1.17)	0.223	0.86 (0.57–1.3)	0.475	0.029	-
		rs2078486	G/A	0.07	0.94 (0.72–1.22)	0.628	0.99 (0.76–1.3)	0.968	0.063	-

1 ch: chromosome; SNP: single nucleotide polymorphism.

2 A: major allele, a: minor allele, MAF: minor allele frequency.

3 odds ratio (95% confidence interval) per minor allele adjusted for age and gender.

4 mediation proportion, calculated only for those with significant total and indirect effect, based on standardized coefficients.

*statistical significance: False Discover Rate (FDR) q-value<0.05^#^ nicotinic receptor genes.

**Table 3:** Results of mediation analyses of SNPs, nicotine dependence (ND) and lung adenocarcinoma (ADC).

			SNP associatedwith ND^2^		ND associated withlung ADC^2^		SNP associatedwith lung ADC^2^	
Chr^1^	gene	SNP^1^	a coef	p-value	b coef	p-value	c′ coef	p-value
5	TERT	rs2736122	0.012	0.889	1.107	**8.1×10^−24^** *	0.014	0.868
		rs4975605	−0.159	0.033	1.090	**7.9×10^−23^** *	−0.028	0.686
		rs2736100	−0.063	0.403	1.107	**1.3×10^−23^** *	−0.272	**1.2×10^−4^** *
		rs2853676	0.028	0.722	1.106	**1.0×10^−23^** *	0.159	**0.031** *
5	CLPTM1L	rs402710	0.033	0.690	1.103	**3.2×10^−23^** *	−0.022	0.774
		rs10073340	0.002	0.987	1.108	**7.4×10^−24^** *	−0.003	0.973
		rs401681	−0.113	0.139	1.105	**1.2×10^−23^** *	−0.173	**0.015** *
		rs31489	−0.062	0.417	1.106	**1.0×10^−23^** *	−0.161	**0.024** *
8	CHRNB3	rs6474414	−0.204	0.029	1.102	**1.5×10^−23^** *	−0.103	0.222
		rs7012713	−0.138	0.511	1.107	**8.2×10^−24^** *	−0.158	0.416
8	CHRNA6	rs892413	−0.015	0.879	1.107	**8.0×10^−24^** *	0.005	0.958
		rs16891604	−0.016	0.908	1.108	**7.4×10^−24^** *	0.002	0.986
		rs16891620	0.055	0.641	1.108	**7.5×10^−24^** *	0.009	0.935
15	CYP1A1	rs4646421	0.273	**0.017** *	1.100	**1.7×10^−23^** *	0.153	0.161
		rs2470893	0.172	0.057	1.107	**9.1×10^−24^** *	0.022	0.797
15	CHRNA5	rs6495306	−0.061	0.437	1.104	**1.2×10^−23^** *	−0.148	**0.043** *
		rs680244	−0.059	0.449	1.102	**1.6×10^−23^** *	−0.151	**0.038** *
		rs621849	−0.056	0.478	1.112	**6.5×10^−24^** *	−0.148	**0.042** *
15	CHRNA3	rs578776	−0.243	**0.006** *	1.088	**6.9×10^−23^** *	−0.309	**1.6×10^−4^** *
		rs12910984	−0.273	**0.003** *	1.093	**5.5×10^−23^** *	−0.353	**4.1×10^−5^** *
		rs1051730	0.241	**0.002** *	1.083	**2.3×10^−22^** *	0.413	**6.9×10^−9^** *
		rs3743077	−0.063	0.420	1.106	**1.1×10^−23^** *	−0.170	**2.0×10^−2^** *
		rs938682	−0.261	**0.005** *	1.086	**9.3×10^−23^** *	−0.361	**2.8×10^−5^** *
		rs12914385	0.211	**0.005** *	1.084	**2.1×10^−22^** *	0.421	**2.4×10^−9^** *
		rs8042374	−0.275	**0.003** *	1.078	**2.0×10^−22^** *	−0.362	**2.6×10^−5^** *
		rs3743075	−0.047	0.565	1.105	**1.1×10^−23^** *	−0.161	**0.034** *
		rs8192475	−0.058	0.769	1.107	**7.9×10^−24^** *	−0.143	0.432
		rs6495309	−0.282	**0.003** *	1.085	**1.0×10^−22^** *	−0.375	**2.0×10^−5^** *
15	CHRNB4	rs1948	−0.056	0.501	1.107	**1.0×10^−23^** *	−0.213	**0.006** *
		rs950776	−0.027	0.745	1.110	**8.0×10^−24^** *	−0.220	**0.005** *
		rs11636753	−0.060	0.455	1.108	**1.1×10^−23^** *	−0.246	**0.001** *
		rs12441998	−0.217	**0.021** *	1.092	**5.2×10^−23^** *	−0.361	**4.7×10^−5^** *
		rs1316971	−0.187	0.045	1.095	**3.5×10^−23^** *	−0.324	**2.3×10^−4^** *
17	TP53	rs12951053	−0.324	0.045	1.113	**5.5×10^−24^** *	0.127	0.355
		rs2909430	−0.063	0.528	1.108	**7.4×10^−24^** *	0.010	0.909
		rs8079544	−0.580	0.025	1.104	**1.2×10^−23^** *	−0.151	0.475
		rs2078486	−0.296	0.059	1.106	**1.1×10^−23^** *	−0.005	0.968

1 ch: chromosome; SNP: single nucleotide polymorphism.

2 logistic regression coefficients (coef) of (1) ND = **a**SNP+e1, and (2) L = **b**ND+**c′**SNP+e3.

*statistical significance: False Discover Rate (FDR) q-value<0.05.

Some genetic variations in *TERT* and *CLPTM1L*, in the region of chromosome 5p15.33, had a direct association with lung ADC risk, but the mediation effect of nicotine dependence was not significant (relationship shown in Figure 1a). Two SNPs in *TERT* (rs2736100 and rs2853676) directly influenced lung cancer risk (OR = 0.76 per T allele, p = 1.2×10^−4^, FDR q = 2.5×10^−4^, and OR = 1.17 per A allele, p = 0.031, FDR q = 0.031, respectively). The indirect effects of these two SNPs in *TERT* on lung cancer risk though nicotine dependence were not significant. In *CLPTM1L*, two SNPs (rs401681 and rs31489) were directly associated with lung ADC risk (OR = 0.84 per T allele, p = 0.015, FDR q = 0.018, and OR = 0.85 per A-allele, p = 0.024, FDR q = 0.026, respectively), but none had a significant mediation effect through nicotine dependence. Thus, most of these SNPs were associated with lung ADC risk though channels other than nicotine dependence. One SNP (rs4646421) in *CYP1A1* was significantly associated with nicotine dependence (p = 0.017, FDR q = 0.043) but its indirect effect on lung ADC became insignificant after multiple comparison justification. The impact of SNPs in another three genes (*CHRNB3, CHRNA6,* and *TP53*) on nicotine dependence and lung ADC was not statistically significant.

## Discussion

To our knowledge, this is the first study to report the significant impact of nicotine dependence on the relationship between genetic variants in the region of *CHRNA5/A3/B4* and lung ADC risk. Most of the SNPs in *CHRNA3* had a significant indirect effect on lung ADC through nicotine dependence. Nicotine dependence is the mediator and contributor of approximately 15% of the association between lung ADC and gene variations of *CHRNA3.* Only variants in the nicotinic receptor genes *(CHRNA5/A3/B4*) had a significant indirect effect on lung ADC through nicotine dependence and variants in the control genes did not. This demonstrated robustness of the mediation analyses in identifying the casual relationship. The significant mediation impact of smoking on the association between rs1051730 in *CHRNA3* and lung related diseases [overall lung cancer and chronic obstructive pulmonary disease (COPD)] were reported previously [22]–[24]. However, the results between these studies and ours are not directly comparable because other studies had different outcomes [lung cancer overall (instead of lung ADC) or COPD] and different mediators (number of cigarettes smoked per day or smoking pack-year). SNP rs1051730 had both a direct effect on overall lung cancer risk and indirect effects through smoking pack-year, and the mediation proportion was 7.6% in ever smokers. This SNP is also significantly associated with COPD, and the mediation proportion through cigarette pack-years is 24% [22]. Two linked variants (rs1051730 and rs8034191) in the *AGPHD1/CHRNA3* cluster are strongly associated with COPD, and the mediation effect is 11–12% through number of cigarettes smoked per day and 26–42% through cigarette pack-years [24]. However, another study based on meta-analyses of four projects had inconsistent results. The impact of two SNPs, rs8034191 and rs1051730, on 15q25.1 on lung cancer through cigarettes smoked per day is not significant [23].

Our findings are consistent with other studies that show that variations in *CHRNA5/A3/B4* are significantly associated with lung ADC risk and nicotine dependence. In our study, the majority of SNPs in the *CHRNA5/A3/B4* region are significantly associated with both nicotine dependence and lung ADC risk. As shown in Table 3, the top two *CHRNA3* SNPs (rs1051730 and rs12914385, r^2^ = 0.93) associated with lung ADC risk also had significant indirect effects through nicotine dependence (***a*** coefficient, p = 0.002 and 0.005, respectively). Many previous studies show that genetic variations of *CHRNA5/A3/B4* relate to overall lung cancer [16], [44]–[46], lung ADC [32], [45], smoking quantities [16], [44], and nicotine dependence [44]. In a meta-analysis study combing ten studies [32], two *CHRNA3* SNPs (rs1051730 and rs12914385) were shown to be significantly associated with lung ADC (p = 7.1×10^−19^ and 3.3×10^−18^, respectively). The impact of these two *CHRNA3* SNPs on lung ADC risk was only significant in ever smokers [32]. Two SNPs (rs1051730 and rs8034191, both with p<1×10^−17^) with strong LD in the regions of 15q25.1 containing *CHRNA3, CHRNA5,* and *PSMA4* are strongly associated with lung cancer risk among ever smokers [46]. SNP rs1051730 in the *CHRNA3* gene was strongly associated with lung cancer risk (p = 1.5×10^−8^) and cigarettes per day (P = 5×10^−16^) [44]. Based on two GWA meta-analyses, rs1051730 is also strongly associated with the number of cigarettes smoked per day for Liu JZ’s study [47] (p = 1.7×10^−66^) and the Tobacco and Genetics Consortium study [48] (P = 2.8×10^−73^). The haplotype A_C of rs16969968 and rs680244 in the region of *CHRNA5/A3/B4* is associated with larger quantities of cigarettes smoked per day and later age at smoking cessation [49]. The three genes of *CHRNA5/A3/B4*, encoding nicotine acetylcholine receptor subunits (α5, α3 and β4) are in the region of chromosome 15q24-25.1. The nicotinic acetylcholine receptor belongs to the superfamily of ligand-gated ion channels, and it is activated by acetylcholine, choline, and nicotine. These proteins are involved in the regulation of nicotine and tobacco nitrosamines, which are the main carcinogens responsible for smoking related lung cancer [50]. In addition, the nicotinic acetylcholine receptors are involved in several pathways and influence cancer progression, such as cell proliferation, apoptosis, invasion, and angiogenesis [51], [52]. Schuller *et al* (2009) suggested that carcinogenesis may be triggered by regulating a complex network of neurotransmitters by altered signaling of the nicotinic acetylcholine receptors [53].

Among our four ‘control’ genes, gene variations in *TERT* (rs2736100 and rs2853676) and *CLPTM1L* (rs401681 and rs31489) have a significant direct association with lung ADC risk but not an indirect effect through nicotine dependence. This means that the association between the SNPs in these two genes is not explained by nicotine dependence but could be explained by other, unmeasured factors. The identified associations between genetic variations in *TERT* and *CLPTM1L* and lung ADC are consistent with previous studies. In a large-scale GWA study [54], rs2736100 in *TERT* associated with lung cancer reached GWA significance (p = 4×10^−6^) and was replicated in an independent set. The significant association between rs2736100 and lung ADC is also reported in a study combining several GWA studies and meta-analyses [32]. In *CLPTM1L*, rs402710 (p = 2×10^−7^), rs31489 (p = 8×10^−7^), and rs401681 (p = 2×10^−6^), were associated with lung cancer risk [54]. Two of these SNPs (rs401681 and rs31489) in *CLPTM1L* are associated with lung cancer risk in ever smokers (p = 1×10^−3^; and p = 2.1×10^−3^)[55]. Genetic variations in *TERT* and *CLPTM1L* at chromosome 5p15.33 are associated with lung ADC risk in smokers [32] and never smokers [55]. However, studies evaluating the association between variations in *TERT* and *CLPTM1L* and smoking behavior are sparse. The *TERT* is the reverse transcriptase component of telomerase. The role of telomeres is to preserve the integrity of the genome during cellular replication [56]. Telomere shortening often leads to chromosomal instability, mutagenesis [57], tumorigenesis [58]–[65], and progression of cancer [66]–[70]. Telomere length may be considered as a biological regulator and a predictive indicator of disease risk, progression, and premature mortality [71]. We recently reported significant associations between changes of telomere lengths and cancer risk [72], [73]. Like the *TERT* gene, *CLPTM1L* is also located at 5p15.33, which is a susceptible region for various cancers, including lung cancer [74]. The *CLPTM1L* was originally identified among the genes involved in resistance to the anticancer agent cisplatin in cancer cell lines [75]. Overexpression of *CLPTM1L* mRNA has been observed in all cisplatin-resistant cell lines examined. However, *CLPTM1L* over-expression doesn’t seem to have any effect on cisplatin-resistant cells and cause apoptosis in cisplatin-sensitive cell lines. Although the exact function of the *CLPTM1L* is not known, it appears that there is an association with resistance to cisplatin and activation of the mitochondrial apoptotic pathway. *CLPTM1L* was expressed in lung tumor tissue, most intensely in ADC tissue, especially in the mitochondria [74]. *CLPTM1L* expression was strongly associated with the tumor grades of differentiation but not smoking status [74].

Translating these findings of genetic variants in the *CHRNA5/A3/B4* region into public health practice may be used to identify high risk groups and could lead to tailored smoking cessation interventions. For individuals with high-risk genetic variants, which have both direct and indirect effects through nicotine dependence on lung ADC, a customized intervention can be applied to assist them to quit smoking and then reduce risk of developing lung ADC. A recent smoking cessation trial in heavy smokers (smoked ≥10 cigarettes per day) shows that those with the high-risk haplotype (A_C allele of rs16969968 and rs680244) in the *CHRNA5/A3/B4* region are biologically predisposed to difficulty in quitting but tended to respond better to pharmacotherapy treatment of smoking cessation than those with low-risk haplotype [49].

The study limitations include self-reported nicotine dependence measurements and a limited sample size. Although self-reported nicotine dependence score based on the Fagerstrom Test [28], which is easy to obtain in practice, is strongly associated with nicotine withdrawal [31], it may not completely capture smoking intensity and concomitant carcinogens. Other objective smoking phenotypes [such as NNK (a nicotine metabolite) and puff volume] have been recommended [76], [77]. Larger and follow-up studies for Whites and other race groups, and an objective smoking phenotype, are warranted to further test the potential applications. In addition, it has been shown that there may be a potential bias in estimating the association between the exposure and the mediator (indirect effects) because the binary mediator is not selected using the principals of a case-control study design [78]–[80]. For adjusting for this bias, the sampling weighting approach has been suggested [23], [81]. Our study did not apply this sampling weighting approach because it is a challenge for obtaining the prevalence of lung ADC in ever smokers in Italy. In summary, our results show that genetic variants in the *CHRNA5/A3/B4* region may impact lung ADC through nicotine dependence. These identified SNPs information may help to detect lung ADC at earlier stages and provided promising support for genetic-guided smoking cessation and lung cancer prevention.

## Supporting Information

Table S1 Indirect effects and their bootstrap confidence intervals of SNPs on lung adenocarcinoma through nicotine dependence.
(DOCX)
