# Supplementary material for: Role of Nicotine Dependence on the Relationship between Variants in the Nicotinic Receptor Genes and Risk of Lung Adenocarcinoma
Source: PLoS One. 2014 Sep 18;9(9):e107268. doi: 10.1371/journal.pone.0107268 (PMC4169410; doi:10.1371/journal.pone.0107268)
Supplement: Table S1 — Indirect effects and their bootstrap confidence intervals of SNPs on lung adenocarcinoma through nicotine dependence. (DOCX) [file pone.0107268.s001.docx]

Table S1. Indirect effects and their bootstrap confidence intervals of SNPs on lung adenocarcinoma through nicotine dependence

| Chr^1^ | Gene | SNP^1^ | IND^2^ | Bootstrap 95% CI^3^ |
| --- | --- | --- | --- | --- |
| 5 | TERT | rs2736122 | 0.001 | (-0.014,0.014) |
|  |  | rs4975605 | -0.016 | (-0.031,-0.002) |
|  |  | rs2736100 | -0.006 | (-0.022,0.009) |
|  |  | rs2853676 | 0.003 | (-0.012,0.016) |
| 5 | CLPTM1L | rs402710 | 0.003 | (-0.012,0.017) |
|  |  | rs10073340 | 0.000 | (-0.015,0.014) |
|  |  | rs401681 | -0.011 | (-0.028,0.004) |
|  |  | rs31489 | -0.006 | (-0.022,0.008) |
| 8 | CHRNB3 | rs6474414 | -0.017 | (-0.033,-0.002) |
|  |  | rs7012713 | -0.005 | (-0.022,0.01) |
| 8 | CHRNA6 | rs892413 | -0.001 | (-0.016,0.012) |
|  |  | rs16891604 | -0.001 | (-0.016,0.013) |
|  |  | rs16891620 | 0.003 | (-0.013,0.018) |
| 15 | CYP1A1 | rs4646421 | 0.016 | (0.003,0.031) |
|  |  | rs2470893 | 0.014 | (0,0.028) |
| 15 | CHRNA5 | rs6495306 | -0.006 | (-0.021,0.008) |
|  |  | rs680244 | -0.006 | (-0.021,0.008) |
|  |  | rs621849 | -0.005 | (-0.02,0.009) |
| 15 | CHRNA3 | rs578776 | -0.021 | (-0.038,-0.006) |
|  |  | rs12910984 | -0.023 | (-0.04,-0.008) |
|  |  | rs1051730 | 0.022 | (0.008,0.036) |
|  |  | rs3743077 | -0.006 | (-0.021,0.008) |
|  |  | rs938682 | -0.022 | (-0.039,-0.007) |
|  |  | rs12914385 | 0.019 | (0.005,0.033) |
|  |  | rs8042374 | -0.023 | (-0.04,-0.008) |
|  |  | rs3743075 | -0.004 | (-0.019,0.009) |
|  |  | rs8192475 | -0.002 | (-0.017,0.012) |
|  |  | rs6495309 | -0.023 | (-0.039,-0.009) |
| 15 | CHRNB4 | rs1948 | -0.005 | (-0.021,0.009) |
|  |  | rs950776 | -0.002 | (-0.018,0.012) |
|  |  | rs11636753 | -0.006 | (-0.021,0.008) |
|  |  | rs12441998 | -0.018 | (-0.034,-0.004) |
|  |  | rs1316971 | -0.015 | (-0.031,-0.002) |
| 17 | TP53 | rs12951053 | -0.016 | (-0.035,-0.001) |
|  |  | rs2909430 | -0.005 | (-0.02,0.01) |
|  |  | rs8079544 | -0.019 | (-0.041,-0.004) |
|  |  | rs2078486 | -0.015 | (-0.035,0) |

^1^ ch: chromosome; SNP: single nucleotide polymorphism

^2^ Indirect effect based on standardized coefficients (**a_s_** x **b_s_**)

^3^ bootstrap 95% confidence interval based on 2000 bootstrap samples
